# Online monitoring of immunoaffinity-based depletion of high-abundance blood proteins by UV spectrophotometry using enhanced green fluorescence protein and FITC-labeled human serum albumin

**DOI:** 10.1186/1477-5956-8-62

**Published:** 2010-12-01

**Authors:** Kyunggon Kim, Jiyoung Yu, Hophil Min, Hyunsoo Kim, Byungwook Kim, Hyeong Gon Yu, Youngsoo Kim

**Affiliations:** 1 Department of Biomedical Sciences, Seoul National University, College of Medicine, 28 Yongon-Dong, Seoul 110-799, Korea; 2 Department of Biomedical Engineering, Seoul National University, College of Medicine, 28 Yongon-Dong, Seoul 110-799, Korea; 3 Department of Ophthalmology, Seoul National University, College of Medicine, 28 Yongon-Dong, Seoul 110-799, Korea; 4 Cancer Research Institute, Seoul National University College of Medicine, 28 Yongon-Dong, Seoul 110-799, Korea

## Abstract

**Background:**

The removal of high-abundance proteins from plasma is an efficient approach to investigating flow-through proteins for biomarker discovery studies. Most depletion methods are based on multiple immunoaffinity methods available commercially including LC columns and spin columns. Despite its usefulness, high-abundance depletion has an intrinsic problem, the sponge effect, which should be assessed during depletion experiments. Concurrently, the yield of depletion of high-abundance proteins must be monitored during the use of the depletion column. To date, there is no reasonable technique for measuring the recovery of flow-through proteins after depletion and assessing the capacity for capture of high-abundance proteins.

**Results:**

In this study, we developed a method of measuring recovery yields of a multiple affinity removal system column easily and rapidly using enhanced green fluorescence protein as an indicator of flow-through proteins. Also, we monitored the capture efficiency through depletion of a high-abundance protein, albumin labeled with fluorescein isothiocyanate.

**Conclusion:**

This simple method can be applied easily to common high-abundance protein depletion methods, effectively reducing experimental variations in biomarker discovery studies.

## Background

Extracellular body fluids that contain plasma and serum are the most valuable sources for biomarker discovery and application, but useful protein biomarkers exist at low concentrations (ng/ml or lower) in blood; approximately 30 high-abundance proteins constitute ~99% of the total protein mass in human plasma, rendering the detection of low-abundance proteins difficult. Several methods have been developed to deplete high-abundance proteins from blood, most of which are based on the immunoaffinity of antibodies toward high-abundance proteins, for example, multiple affinity removal system (MARS, Agilent, CA, USA) columns which can remove several specific high-abundance proteins [1-5], but some reports have demonstrated that these systems incur problems, such as incomplete removal of high-abundance proteins [3,6,7]. Recently, Bellei at al. showed that untargeted proteins are removed concomitantly [8] and Chengjian et al. also reported that untargeted human plasma proteins, such as alpha-1-acid glycoprotein 1, alpha-1-acid glycoprotein 2, and alpha-1-antichymotrypsin, bind to MARS columns [9].

In fact, the reproducibility of the removal and recovery of flow-through proteins is a critical aspect of high-abundance protein depletion and although Veronica et al. reported that in a triplicate 2-DE experiment, the coefficient of variation (C.V.) of flow-through and bound fractions by MARS depletion was 5.31% and 4.12%, respectively, 3 depletions were not sufficient to validate the reproducibility with regard to column life (200 times is the minimum, as specified in the product manual of the MARS column) [10]. Chengjian et al. observed that after triplicate depletion of plasma, the capture efficiency of high-abundance proteins exceeded 99% and that 12 nontarget proteins were captured by the MARS column--in particular, 100% of hemoglobin subunit alpha and subunit beta were captured. Of the 12 nontarget proteins that were reported in Chengjian's study, several have important biological functions, such as alpha-1-antichymotrypsin, which mediates rheumatoid arthritis [11]. Therefore, capture efficiency and nonspecific binding events should be monitored periodically or, if needed, during every run. Yet, there is no convenient quality monitoring process that monitors such parameters. Several techniques, such as bicinchoninic acid (BCA) assay and enzyme-linked immunosorbent assay (ELISA), have been used to determine the extent of depletion [7,12], but post depletion experiments require significant funds and time. Specifically, in biomarker discovery or verification processes, hundreds of patient plasma samples should be analyzed. If there is no effective quality monitoring step during the depletion of hundreds of plasma samples, the resulting variations in depletion can generate significant errors. One can determine approximately whether the depletion of plasma has been performed correctly by observing the UV chromatogram of LC at 280 nm, but this chromatogram does not yield accurate data on nontarget protein binding or target protein capture. Enhanced green fluorescence protein (EGFP) is a mutant of green fluorescence protein (GFP), which has a maximum excitation wavelength of 488 nm [13-15]. Its fluorescence is proportional to its quantity, allowing one to measure its concentration solely by measuring the UV absorbance. We monitored the UV absorbance of several proteins, including EGFP, to assess the performance and reproducibility of the depletion for high-abundance proteins in blood

In this study, EGFP was expressed in an *E. coli *protein expression system and purified, and a known amount of EGFP was spiked into a clinical sample, such as plasma or serum. During the depletion of high-abundance proteins from plasma or serum, EGFP is an indicator of flow-through proteins out of the MARS depletion column. EGFP exists stably in plasma, and EGFP monitoring by UV absorbance at 488 nm (UV_488 nm_) provides quality monitoring data on reproducibility and recovery during depletion. In addition, one must track not only the flow-through proteins but also the high-abundance proteins that bind the antibody-based depletion column. According to data from Agilent, buffer B, which is used in the elution step, is based on urea; thus, Agilent has specified that column performance is maintained up to 200 uses. Thus, during repeated use, depletion columns capture fewer high-abundance proteins, and the life of the column varies, depending on handling. Further, column performance cannot be monitored online. Routine SDS-PAGE and Western blot or ELISA can be performed to determine the efficacy of depletion. These processes, however, take time and are costly.

In this study, we labeled human serum albumin (HSA), the most abundant protein in plasma, with fluorescein isothiocyanate (FITC) to monitor the capacity of a depletion column. A known amount of FITC-labeled HSA (FITC-HSA) was spiked into depleted plasma or depleted serum as an indicator of high-abundance proteins. Because FITC also has a maximum excitation at 488 nm, we measured the depletion efficiency using EGFP for flow-through proteins and FITC-HSA for high-abundance proteins by online monitoring at UV_488 nm _during an HPLC run. Thus, we developed a convenient method for measuring: 1. the recovery of flow-through proteins by monitoring EGFP and 2. the removal of high-abundance proteins using FITC-HSA at UV_488 nm _during depletion. This online monitoring system conveniently tracks the recovery and performance of all depletion systems that are run by HPLC, including the MARS depletion column. In biomarker discovery or verification steps, periodical online monitoring using EGFP or FITC-HSA assesses the quality of depletion runs and identifies unsuitable cases from the depletion runs, which can reduce experimental variation.

## Materials and methods

### Reagents

MARS columns (4.6 mm × 10 mm), solution A, and solution B (Product No. 5185-5985) were provided from Koreabiomics, a distributor for Agilent in Korea. There are limited data on solutions A and B, which are based on water with sodium azide and urea, respectively. For FITC labeling, fluorescein isothiocyanate (FITC, Cat. No. F7250-1G) and human serum albumin (HSA, Cat. No. A1653-10G) were purchased from Sigma. PCR primers for EGFP were synthesized by CosmoGentech (Seoul, Korea). LB media powder was purchased from Biopure (Cat. No: LB407, Canada). Urea for 2DE-PAGE was purchased from Amresco (Code: 0568-1KG, Ohio, USA), and thiourea was obtained from GE Healthcare (Cat. No.: RPN6301V, UK). Sodium carbonate for FITC labeling was purchased from Amresco (Code: 0585-500G), and sodium thiosulfate was purchased from Sigma (Cat. No.: S-7026). Other reagents that are not mentioned here were obtained from Sigma.

#### Plasma sample preparation

Human plasma samples were provided by Seoul National University Hospital. All individuals provided informed consent before being enrolled in this study, in accordance with the approved protocol of the Institutional Review Board at Seoul National University Hospital. To reduce sampling bias due to individual variations, 100-μl plasma samples from 10 healthy persons (5 females and 5 males) were pooled and centrifuged at 22,250 *g *at 4°C for 10 minutes to remove debris. The centrifuged plasma was stored in 50-μl aliquots at -80°C until use. For the depletion experiments, the plasma was thawed at 4°C.

#### Expression of enhanced green fluorescent protein

The sequence of EGFP was obtained from the Lablife website http://www.lablife.org. The entire EGFP coding sequence (residues 1-231) was generated by PCR using the vector pEGFP N1 (Clonetech, CA, USA) with the sense primer 5'- GAC*AAGCTT***AT**ATGGTGAGCA-3' and the antisense primer 5'-GCCGGGATCACT*CTCGAG*CAC-3'. The underlined italic letters denote *Hind*III and *Xho*I restriction sites, respectively, and the bold letters are additional bases that generated an open-reading frame. After digestion with each restriction enzyme, gel extraction was performed, followed by ligation into the prepared pET 24a (+) vector (Novagen, WI, USA).

pET24a(+)-EGFP-His was transformed into BL21 RIL codon-plus cells (DE3) (Novagen), and overnight culture seeds of BL21-CodonPlus (DE3)-RIL that harbored the EGFP DNA construct (5 ml) were used to inoculate 200 ml LB medium, containing kanamycin (30 μg/ml), and grown overnight at 37°C. When the optical density of the cells at 600 nm reached 1.0, protein expression was induced by adding 0.5 mM isopropyl β-d-thiogalactopyranoside (IPTG) at 21°C.

After overnight induction, cells were harvested by centrifugation at 5,000 *g *for 10 min. Cell pellets were resuspended in ice-cold 20 mM Tris-HCl (pH 7.8), containing 200 mM NaCl, 1 mM β-mercaptoethanol (βME), and protease inhibitor cocktail (Roche, Switzerland). Resuspended cells were lysed by ultrasonication. Cell supernatants were generated by centrifugation at 15,000 *g *for 20 min at 4°C. EGFP was purified by Ni-NTA purification. Amounts of EGFP were measured by BCA analysis and UV_488 nm _absorbance at the same concentrations.

#### Determination of EGFP standard curve using UV_488 nm_

To construct a standard curve for EGFP, EGFP was serially diluted from 0 to 0.6 mg/ml (increments of 0.1 mg/ml, 7 points) in MARS buffer A (Agilent, CA, USA). Two hundred microliters of each diluted sample (blank, 20, 40, 60, 80, 100, and 120 μg EGFP) was transferred to a 96-well plate (SPL, Korea) and read from 400 nm to 600 nm on an ELISA reader (PowerWave XS, Bio-Tek, VT, USA); the standard curve was generated from known amounts of EGFP, and the observed absorbance at 488 nm was calculated by linear regression and *R*^2^.

#### FITC-labeling of human serum albumin

One hundred micrograms of HSA (1.5 μmol) (MW: 66,472 Da, mature form) was dissolved in 20 ml of 100 mM sodium carbonate (pH 9.3), and 1.5 mg of FITC (3.85 μmol) (M.W: 389.38 Da) was dissolved in 500 ml dimethyl sulfoxide (DMSO, Cat. No: C6295, Sigma). The solutions were mixed at a 1:2.56 molar ratio (HSA: FITC = 1.5 μmol: 3.85 μmol) and incubated overnight at 4°C.

Solutions of FITC-HSA and unlabeled FITC were concentrated using a 5-kDa cutoff Centricon filter (Millipore, MA, USA), dialyzed in Slide-A-Lyzer dialysis cassettes (Pierce, Rockford, IL, USA) in 1 L PBS 5 times (the final dialysis was done in 1 L PBS overnight), and reconcentrated on a 5-kDa cutoff Centricon filter (Millipore) to remove unlabeled FITC. FITC-HSA was concentrated to 500 μl and purified on a Superdex S-200 gel filtration column by AKTA FPLC (Amersham Biosciences, Uppsala, Sweden) to enhance the purity. A 30-cm Tricon column (internal diameter: 10 mm, GE Healthcare) was used at a flow rate of 0.3 ml/min. PBS was used as the buffer for size-exclusion chromatography. After gel filtration, the eluted fraction of pure FITC-HSA was pooled, concentrated to 1 mg/ml, and stored in the dark at 4°C until use.

#### Depletion of human plasma using MARS column chromatography

To deplete plasma using the MARS column, plasma was diluted 5-fold with MARS buffer A and filtered on a 0.22-μm Ultrafree-MC Durapore centrifugal filter (Millipore, Cat. No: UFC30GVNB). Plasma only, EGFP-spiked plasma, and EGFP-only samples were applied to a MARS column on an LC-10AT HPLC system (Shimadzu, Kyoto, Japan), as described below. The sample loop volume of HPLC was set to 100 μl, wherein 58 μl of 5-fold diluted plasma (total weight 2.32 mg) and 42 μl EGFP (final concentration of 0.3 mg/ml, total weight 300 μg) for EGFP-spiked plasma or 58 μl MARS buffer A and 42 μl EGFP (final concentration of 0.3 mg/ml, total weight of 300 μg) for EGFP-only were injected into the MARS column.

The total LC run time of 45 min comprised the following sequence: 100% MARS buffer A at a flow rate of 0.7 ml/min for an initial 15 min, sample injection, a 15-min wash, 100% MARS buffer B at a flow rate of 1.0 ml/min for 5 min, and 100% MARS buffer A at a flow rate of 0.7 ml/min for 10 min. The UV detector was set to 280 nm for the injection of plasma only or 488 nm for the injection of EGFP-spiked plasma or EGFP-only samples, and the eluted fractions were collected in 500 μl. The flow-through and bound fractions were eluted in 5 (total 2.5 ml) and 10 vials (total 5 ml), respectively, for which the 2 eluted fractions were pooled, respectively. The 2 fractions were reduced to 1 ml on a Centricon. After determining the concentration of the fractions by BCA assay, the fractions was subjected to Western blot, 1-DE, or 2-DE PAGE. The resulting UV chromatograms were analyzed to determine peak areas and retention times.

To determine whether FITC-HSA, as an indicator of high-abundance proteins was separable on a MARS column, 600 μg FITC-HSA was injected into a MARS column in triplicate while the UV detector was set to 488 nm. In addition, the depletion of FITC-HSA from plasma was examined, wherein 600 μg FITC-HSA was spiked into HSA-depleted plasma to simulate HSA-undepleted plasma. The HSA-depleted plasma that was spiked with FITC-HSA was injected into a MARS column in triplicate with the UV detector set to 488 nm. The resulting UV chromatograms were analyzed to calculate peak areas and retention times.

#### Calculation of EGFP concentration to determine the recovery of depletion

For plasma depletion, the volumes of the unbound fraction containing EGFP and the bound fraction were 2.5 ml and 5.0 ml, respectively. Each fraction was pooled and concentrated to 1.0 ml on a Nanosep 3K centrifugal filter (Pall Corporation, NY, USA). Next, 200 μl of the concentrated samples was transferred to 96-well plates (SPL, Korea) to measure UV absorbance at 488 nm on an ELISA reader, and the UV absorbance was extrapolated on the standard curve to determine EGFP concentration.

#### 2-dimensional gel electrophoresis and silver staining

After plasma depletion, bound fractions that contained high-abundance proteins were pooled and buffer-exchanged with 5 ml of rehydration buffer (7 M urea, 2 M thiourea, 2% CHAPS, 60 mM DTT, and 0.5% (v/v) pharmalyte (pH 3-10)) 5 times to remove any traces of buffer B. After protein concentration was determined by Bradford assay, 30 μg of bound fraction was subjected to IEF using immobilized pH gradient (IPG) strips (7 cm, pH 4-7, linear gradient; Amersham Biosciences, Sweden), as described [16]. Briefly, isoelectric focusing was carried out on an IPG strip for 10,500 volt-hour at 20°C (500 volt in 1 hour, 4000 volts in 2.5 hours) using an IPGphore system (Amersham Biosciences, Uppsala, Sweden). Then, the strips were equilibrated for 30 min in reducing solution (50 mM Tris-HCl (pH 8.8), 6 M urea, 30% (v/v) glycerol, 2% (w/v) SDS, 1% (w/v) DTT) and equilibrated for 30 min in alkylating solution, which was the reducing solution plus 2.5% (w/v) iodoacetamide.

Gel electrophoresis in the second dimension was performed using a standard SDS-PAGE protocol. After SDS-PAGE, protein spots were visualized by silver staining as described [17]. Briefly, after SDS-PAGE, the gel was placed into fixation solution (40% MeOH, 10% acetic acid) for 40 min and washed in 30% EtOH solution. After a sensitizing step with 0.02% sodium thiosulfate for 1 minute, the gel was washed twice in distilled water for 30 sec. Silver reactions was performed with 0.2% silver nitrate and 0.02% formaldehyde (37% w/w) solution for 20 min, and the gel was washed twice in distilled water for 2 min. The gel was developed in 3% sodium carbonate and 0.05% formaldehyde (37% w/w) for 3 min and stopped using 0.5% glycine solution. The stained gel was scanned on a UMAX PowerLook 2100 XL (UMAX Technologies, TX). The spots in the 2-DE gel image was cut and in-gel digested; MALDI-TOF/TOF (Model: 4700 Proteome Discovery, AppliedBiosystems, Foster city, USA) analysis was performed to confirm the proteins as described (data not shown) [18].

#### Western blot analysis

For Western blot analysis, the concentrated bound fraction in MARS buffer B was buffer-exchanged with 1 ml MARS buffer A 10 times on a Nanosep 3K centrifugal filter; MARS buffer B reacts with BCA solution to generate color. After BCA analysis, 5 μg was separated on 12% SDS-PAGE gels and transferred to PVDF membranes at 100 V (GE Healthcare, NJ, USA) for 1 hr at 4°C.

The membranes were blocked with 5% (w/v) BSA, 0.1% Tween 20 in TBS buffer for 2 hr at room temperature and incubated with primary antibodies on a shaker at 4°C overnight. The primary antibodies were mouse anti-human transferrin (1:1000; AbFrontier), mouse anti-human alpha-1-antitrypsin (1:1000; AbFrontier), mouse anti-human haptoglobin (1:1000; AbFrontier), and rabbit anti-his probe (2:1000; Santa Cruz Biotechnology, CA, USA).

After being washed in 0.5% Tween 20 in TBS for 10 min 5 times, the membranes were incubated with horseradish peroxidase-conjugated secondary antibodies (goat anti-mouse IgG-HRP for transferrin, alpha-1-antitrypsin, and haptoglobin; and goat anti-rabbit IgG-HRP for EGFP-His, Santa Cruz) at 1:5000 for 1 hr at room temperature. Signals were detected using ECL Plus Western Blotting Detection Reagents (GE Healthcare) on an LAS-4000 Image analyzer (Fujifilm, Tokyo, Japan). Signals were analyzed quantitatively using image analysis software (2D Phoretix expression v2004, Newcastle, UK). All results were expressed as means and analyzed statistically by Student's *t*-test.

## Results and Discussion

**Figure 1:**
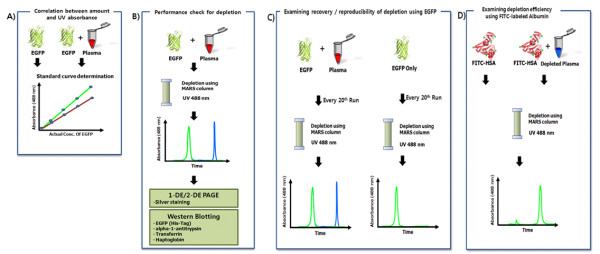
**Schematic of experiments**. (A) In the first stage, EGFP was purified, and the correlation between the quantity of EGFP and UV absorbance at 488 nm was calculated. From these data, a standard curve was generated. (B) 300 μg EGFP was spiked into 2.21 mg of 5-fold-diluted plasma, and MARS depletion was performed under UV_488 nm _monitoring in 6 repeat runs. Unbound and bound fractions were analyzed by SDS-PAGE and 2-DE. In addition, Western blot was performed for EGFP, alpha-1-antitrypsin, transferring, and haptoglobin. The OD at 488 nm was measured to determine the recovery of EGFP from EGFP-only and EGFP-spiked plasma. (C) During 200 runs of the MARS column, EGFP-only or EGFP-spiked plasma was injected as an indicator of flow-through proteins for the quality assessment at every 20th run, in which the reproducibility of depletion was calculated. (D) Depletions were performed using FITC-HSA as a high-abundance protein indicator to determine the capture efficiency of high-abundance proteins.

This study is divided into 4 sections, as described in Figure 1. The first section comprises the preparation of EGFP and generation of a standard curve using 7 points of EGFP concentration and its corresponding UV absorbance at 488 nm (Figure 1A). In the second section, plasma that was spiked with 300 μg EGFP as an indicator of flow-through proteins was depleted using a MARS column, and Western blot was employed to examine the performance of EGFP as an indicator (Figure 1B). In the third section, EGFP-only and EGFP-spiked plasma samples were depleted every 20th run in 200 depletion experiments. Recovery of EGFP using EGFP-only and EGFP-spiked plasma was measured using UV absorbance at 488 nm to assess reproducibility throughout the life (200 depletion runs) of a MARS column (Figure 1C). In the fourth section, FITC-HSA, as a high-abundance protein indicator, was spiked into depleted plasma, and high-abundance proteins were depleted to determine the capture capacity of the MARS column. For these 4 steps, commercial MARS columns were used fewer than 200 runs, as recommended (user manual, Agilent).

### EGFP purification and FITC labeling of human serum albumin

**Figure 2:**
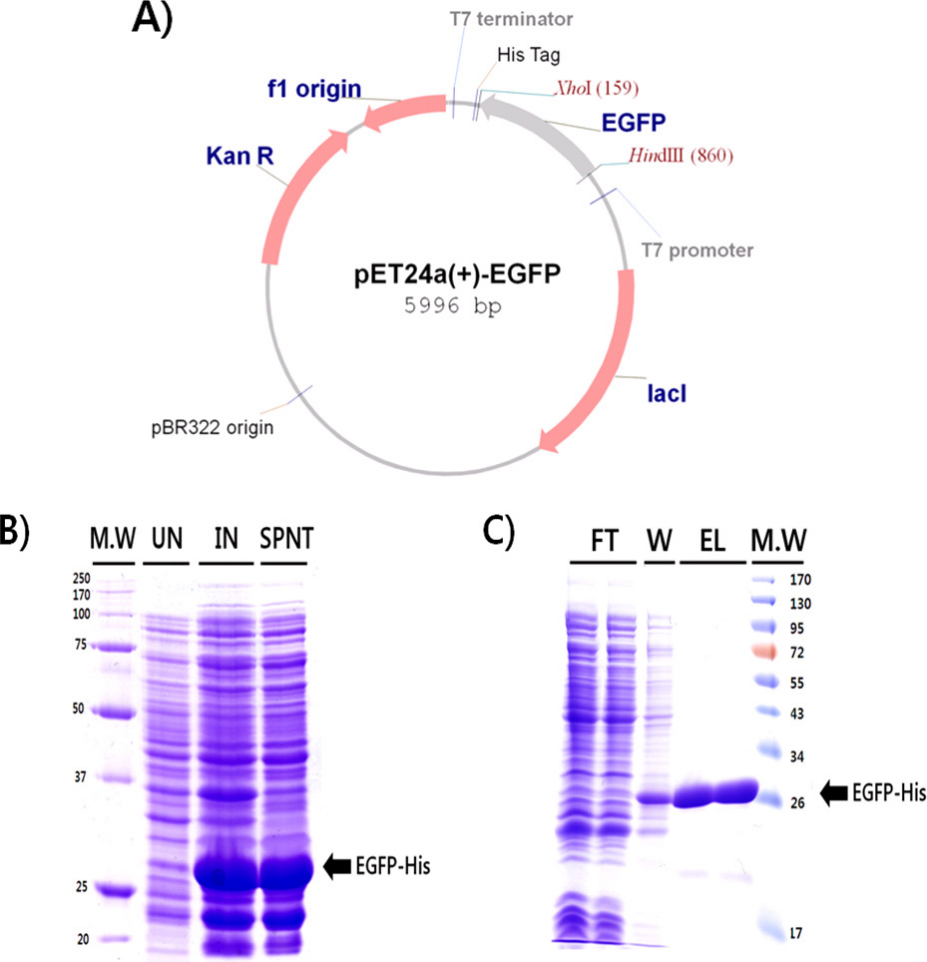
**Cloning, overexpression, and purification of EGFP**. **(A) **Map of pET24a (+)-EGFP-His, drawn with vector NTI software (version 6.0, InforMax). The EGFP gene is designated by the gray arrow. (B) pET24a (+)-EGFP-His was transformed into BL21 RIL codon-plus (DE3) (Novagen) and overexpressed by IPTG induction. Molecular weight marker (MW), uninduced total cell lysate (UN), induced total cell lysate (IN), and induced supernatant (SPNT) were loaded on a 12% SDS-PAGE gel and visualized using Coomassie Brilliant Blue R250. (C) Purification was performed by Ni-NTA, and the flow-through fraction (FT), wash fraction (W), and eluted fraction (EL) were run on a 12% SDS-PAGE gel. The EGFP-His band is indicated with a black arrow.

EGFP, with a C-terminal hexa-histidine tag, was expressed in the BL21 (DE3) codon plus *E. coli *strain. The yield of EGFP was approximately 50 mg/L in LB media, and the purity was high, as shown by SDS-PAGE (Figure 2).

To prepare FITC-HSA, HSA was labeled with FITC at a ratio of 1:2.56, and the labeling efficiency was measured as described in Additional file 1: Supplementary Figure 1. To FITC-label HSA, the OD_280 _and OD_495 _of FITC-HSA and FITC alone were measured; the labeling of HSA with FITC was estimated as described in the FITC labeling protocol [19]. Consequently, the labeling ratio of HSA to FITC was 1:2.61, based on the calculated value (Additional file 1: Supplementary Figure 1), which approximates the input value (1:2.56). Considering measurement errors, FITC labeling of HSA proceeded nearly to completion; thus, additional purification was not needed to separate FITC-HSA from unlabeled HSA. The linearity of FITC fluorescence was examined, based on the amounts of FITC-HSA on increasing FITC-HSA levels, as described in Additional file 1: Supplementary Figure 2. Apparently, FITC fluorescence represented the corresponding amounts of spiked FITC-HAS in the depletion samples.

### Linearity between EGFP concentration and UV absorbance at 488 nm

**Figure 3:**
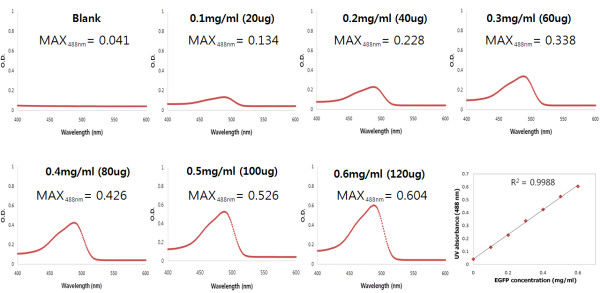
**Linear correlation of UV_488 nm _versus EGFP concentration**. EGFP was diluted from 0.1 mg/ml to 0.6 mg/ml in MARS buffer A in 0.1-mg/ml increments, generating 7 concentration points. The UV absorbance of 200 μl of diluted sample was measured, ranging from 400 nm to 600 nm, on an ELISA reader. (A) Blank (MARS buffer A), (B) 0.1 mg/ml (20 μg), (C) 0.2 mg/ml (40 μg), (D) 0.3 mg/ml (60 μg), (E) 0.4 mg/ml (80 μg), (F) 0.5 mg/ml (100 μg), and (G) 0.6 mg/ml (120 μg). (H) Linear correlation of UV absorbance versus EGFP concentration was calculated by linear regression. The correlation of coefficient (*R*^2^) is 0.9988, as shown in the graph.

EGFP concentration was measured to examine the linearity of EGFP between 0.1 mg/ml and 0.6 mg/ml (0.1-mg/ml intervals) versus UV absorbance at 488 nm (Figure 3). EGFP concentration showed an excellent linear relationship versus UV absorbance at 488 nm, as evidenced by *R*^2 ^= 0.9988 (Figure 3H). The limit of quantitation (LOQ) of EGFP was 100 μg/ml (20 μg). The concentration of EGFP, 300 μg/ml, was chosen for further experiments, because it was in the middle of the standard curve.

### Performance of EGFP as indicator during depletion using EGFP-only or EGFP-spiked plasma

**Figure 4:**
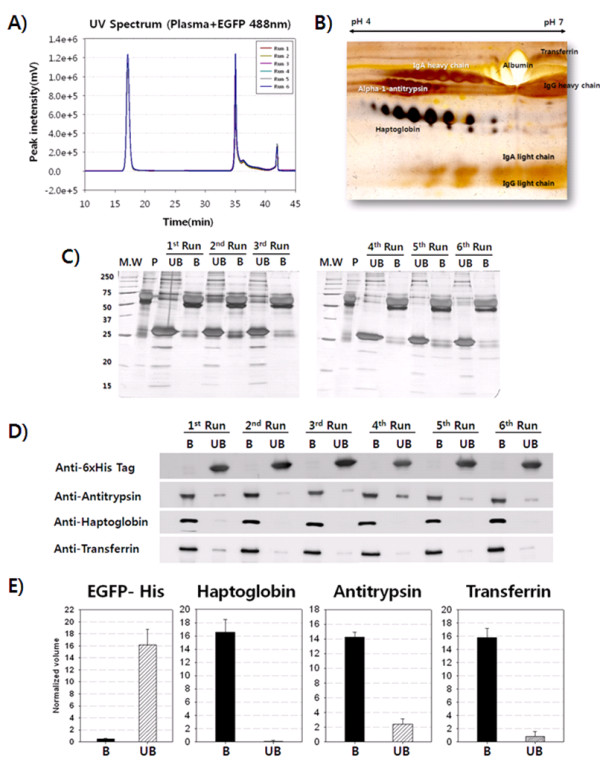
**Performance of EGFP from EGFP-only or EGFP-spiked plasma in depletion experiments**. (A) Overlay of chromatograms from 6 consecutive MARS depletion runs using EGFP-spiked plasma. A mixture of flow-through proteins and EGFP as a flow-through protein indicator were eluted as an unbound fraction at 17 min, and the high-abundance proteins were bound to the MARS column and eluted as the bound fraction at 35 min. (B) Thirty micrograms of bound fraction (high-abundance proteins) was subjected to 2-DE for 7 cm from pH 4 to pH 7. Six high-abundance proteins on a silver-stained gel were visualized, and MALDI-TOF/TOF analysis was performed to identify spots (data not shown). (C) Equal amounts (5 μg) of plasma (P), unbound fraction (UB, flow-through proteins), and bound fraction (B, high-abundance proteins) from the EGFP-spiked plasma depletion were run on an 8-12% SDS-PAGE gel and visualized by silver staining. The thick band at 25 kDa in the UB lane constitutes spiked EGFP; there is no such band in the B lane. (D) The unbound fraction (UB) and bound fraction (B) were analyzed by Western blot to detect target proteins. Anti-6×His tag was used to detect His-tagged EGFP, and antibodies for antitrypsin, haptoglobin, and transferrin were used for the bound and unbound fractions from the 6 consecutive depletion runs. Serum albumin and immunoglobulin were excluded from the Western blot due to their abundance. (E) Each protein in the Western blots was expressed as band intensity and shown on a bar graph with standard errors, using the Phoretix gel analysis program. In the alpha-1-antitrypsin and transferrin results, there were traces of the unbound fraction, which might have been caused by homology to the serpine family and other proteins.

To examine the reproducibility of EGFP as a flow-through indicator in plasma, depletion experiments were performed 6 consecutive times, wherein 58 μl of diluted plasma (2.32 mg), spiked with 42 μl EGFP (300 μg), was applied onto the MARS column. The chromatograms for the 6 runs were reproducible with regard to retention time and UV intensity for EGFP-spiked plasma (Figure 4A). The coefficient of variation (C.V.) was 0.08% for the retention time of the EGFP peak at 17 min (Additional file 1: Table S1).

A high-abundance protein fraction (30 μg) was subjected to 2-DE and visualized by silver staining. In the 2-DE image, 6 high-abundance proteins were observed (Figure 4B), consistent with a previous study [20,21]. The bound and unbound fractions in the 6 depletion experiments were also examined by SDS-PAGE (Figure 4C). The number and shape of the SDS-PAGE bands were identical to those of a previous report [7]. Quantitative analysis using SDS-PAGE band intensity was attempted, but the gaps between the bands by SDS-PAGE were too narrow, and some target bands overlapped with unidentified bands.

Next, we examined the efficacy of EGFP in MARS column depletion by analyzing the bound and unbound fractions of EGFP-spiked plasma. We assessed the efficacy using Western blot by examining whether the MARS column depleted representative high-abundance proteins, such as α1-antitrypsin, haptoglobin, and transferrin, but it did not deplete flow-through proteins, such as His-tagged EGFP (Figure 4D). Three (α1-antitrypsin, haptoglobin, and transferrin) of 6 high-abundance proteins were selected for Western blot analysis, because the silver-stained bands of albumin and immunoglobulin by SDS-PAGE were adequately visible. Anti-His was used to probe for EGFP, because it contained a His-tag for purification purposes. Because EGFP was not subject to depletion by the MARS column, it should have been detected only in the unbound fractions.

As shown in Figure 4D, EGFP was detected only in the unbound fraction; thus, it did not experience the albumin sponge effect [22,23] or carry over to the bound fraction. Haptoglobin was nearly depleted from the unbound fraction, but α1-antitrypsin and transferrin were not depleted completely in the unbound fractions (Figure 4D). A small amount of α1-antitrypsin in the unbound fraction can be inferred due to the many α1-antitrypsin-homologous proteins in the serpine family and the varying specificities of the primary antibodies between suppliers. The intensity of each Western blot band was analyzed and expressed in bar graphs with standard error marks using Phoretix gel analysis software (Figure 4E).

### Reproducibility of depletion using EGFP-spiked plasma and EGFP-only

**Figure 5:**
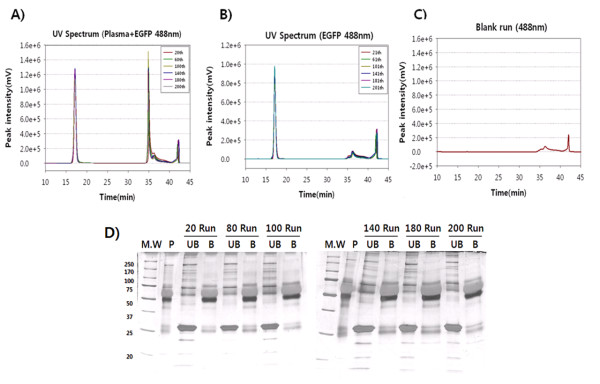
**Recovery using EGFP as a flow-through protein indicator**. (A) Ten chromatograms from the 20th, 60th, 100th, 140th, 180^th^, and 200th MARS column runs using EGFP-spiked plasma were overlain, and an overlay of 10 chromatograms from the 21st, 61st, 101st, 141st, 181st, and 201st MARS column runs using EGFP-only was drawn in (B) using the same pattern. (C) One hundred microliters of MARS buffer A was injected into the MARS column as the blank run. This spectrum at UV_488 nm _generated 2 peaks at 37 min and 43 min from the buffer components. Thus, the 2 peaks were ignored in subsequent experiments. (D) Equal amounts (5 μg) of plasma (P), unbound fraction (UB), and bound fraction (B) from the 20th, 80th, 100th, 140th, 180^th^, and 200th runs from the 10 chromatograms were loaded onto an 8-12% SDS-PAGE gel and visualized by silver staining. The bands at 25 kDa in the UB lane ere spiked EGFP; such bands were not detected in any other B lane.

We determined the reproducibility of depletion with EGFP-only or EGFP-spiked plasma within the recommended use of MARS columns (200 runs). Injection samples were prepared identically in the 6 repeat experiments as in the previous section (300 μg of EGFP and 2.62 mg of plasma protein in 100 μl). EGFP-spiked plasma was injected into the MARS column every 20th run from the 20th to 200th run, and the resulting 10 chromatograms were overlain, as shown in Figure 5A. For the EGFP-only injection, 300 μg EGFP was injected every 20th run from the 21st to 201st run, and the resulting 10 chromatograms were overlain, as shown in Figure 5B.

In these UV chromatograms, the peak of unbound fractions (flow-through proteins) appeared at 17 min, and that of the bound fractions (high-abundance proteins) appeared at 35 min in all 6 batches. The C.V. for the peak area and retention time was 8.5% and 0.25%, respectively, at 17 min (EGFP + other flow-through proteins) during EGFP-spiked plasma depletion (Table 1); the C.V. for the peak area and peak retention time was 5.7% and 0.75%, respectively, at 17 minutes (EGFP) in EGFP-only depletion (Table 2). The C.V. values of the bound fraction in both cases (EGFP-spiked plasma and EGFP-only) were 23.2% and 36%, respectively. The high variation in the bound fraction peak of spiked EGFP was caused in part by a fluctuation in the 4th run, and the variation in the bound fraction peak of EGFP-only was due to instability of the UV detection, because the peak at this retention time was from buffer, not proteins. Two peaks at retention times of 36 min and 42 min also developed in all runs, but they did not harbor proteins, because they were detected in the blank run with an identical peak height and retention time (Figure 5C). All runs in this study confirmed that depletion by the MARS column is reproducible for up to 201 cycles. Figure 5D shows the SDS-PAGE analysis of the 6 depletion experiments after injection of EGFP-spiked plasma for the flow-through and high-abundance protein fractions.

**Table 1:** Recovery of EGFP in EGFP-spiked plasma using EGFP as a flow-through protein indicator

**Run**	**O.D**_**488 nm**_^**a)**^	**EGFP conc. (mg/ml)**^**b)**^	**Recovery (%)**^**c)**^	**Retention Time (min)**	**Peak Area**	**O.D**_**488 nm**_^**d)**^	**Retention Time (min)**	**Peak Area**
**Unbound fraction**						**Bound fraction**		
	
**20**	0.318	0.288	96.108	17.204	41318386	0.042	35.119	21663431
**40**	0.31	0.28	93.291	17.198	41864592	0.042	35.132	20405735
**60**	0.316	0.286	95.404	17.13	46612910	0.043	35.003	27522817
**80**	0.314	0.284	94.7	17.234	48029530	0.043	35.087	40458786
**100**	0.313	0.283	94.348	17.116	47410534	0.042	34.888	29504690
**120**	0.302	0.271	90.474	17.117	42958986	0.042	34.9	24795107
**140**	0.309	0.279	92.939	17.157	44253260	0.042	34.918	23585379
**160**	0.312	0.282	93.995	17.143	37695839	0.043	34.889	21280293
**180**	0.314	0.284	94.7	17.119	38865664	0.042	34.906	21914998
**200**	0.317	0.287	95.756	17.166	39476099	0.042	34.916	25579936
	
**Average**	**0.313**	**0.283**	**94.172**	**17.158**	**42848580**	**0.042**	**34.976**	**25671117.2**
**S.D**.	**0.0047**	**0.0049**	**1.6456**	**0.042**	**3665255.437**	**0.0005**	**0.1**	**5954454.913**
**C.V.(%)**	**1.502**	**1.731**	**1.747**	**0.245**	**8.554**	**1.19**	**0.286**	**23.195**

a) The unbound fractions were concentrated to 1 ml, for which the UV absorbance was measured at 488 nm.

b) EGFP concentration was calculated using the standard curve, as shown in Figure 3H.

c) EGFP recovery (%) = EGFP concentration/0.3 × 100.

d) The bound fractions were concentrated to 1 ml, for which the UV absorbance was measured at 488 nm.

EGFP-spiked plasma (300 μg of EGFP and 2.62 mg of plasma in 100 μl) was depleted every 20th run from runs 20 to 200. Ten chromatograms were analyzed to calculate retention time and peak area. At the same time, unbound fraction and bound fraction from 10 independent depletions were concentrated to 1.0 ml, and OD_488 nm _was measured to calculate recovery. Concentrations of EGFP in the unbound fraction and bound fraction were calculated according to the standard curve, generated in Figure 3. The coefficient of variation (C.V. (%)) was calculated from the standard deviation, divided by the average and multiplied by 100. There was fluctuation in the 4th run, causing increase in C.V. (%) in the bound fraction peak area.

**Table 2:** Recovery of EGFP from EGFP-only solution using EGFP as a flow-through protein indicator

**Run**	**O.D**_**488 nm**_^**a)**^	**EGFP conc. (mg/ml)**^**b)**^	**Recovery (%)**^**c)**^	**Retention Time (min)**	**Peak Area**	**O.D**_**488 nm**_^**d)**^	**Retention Time (min)**	**Peak Area**
**Unbound fraction**						**Bound fraction**		
	
**21**	0.323	0.294	97.869	17.176	27072418	0.042	36.265	12098039
**41**	0.296	0.265	88.361	17.158	25303785	0.042	36.316	12924057
**61**	0.314	0.284	94.7	17.128	26898491	0.043	36.408	7177513
**81**	0.316	0.286	95.404	17.229	27372996	0.043	36.665	5887288
**101**	0.313	0.283	94.348	17.137	27712834	0.042	36.387	5351547
**121**	0.309	0.279	92.939	17.142	25688223	0.042	36.22	8400685
**141**	0.307	0.277	92.235	17.154	27835164	0.043	36.146	9345686
**161**	0.31	0.28	93.291	17.152	23774802	0.043	36.106	5814103
**181**	0.313	0.283	94.348	17.124	27535453	0.042	36.149	5474879
**201**	0.314	0.284	94.7	17.139	27848972	0.041	36.216	5325185
	
**Average**	**0.312**	**0.281**	**93.819**	**17.154**	**26704313.8**	**0.042**	**36.288**	**7779898.2**
**S.D**.	**0.007**	**0.0074**	**2.4582**	**0.03**	**1353730.325**	**0.0007**	**0.167**	**2847817.672**
**C.V. (%)**	**2.244**	**2.633**	**2.62**	**0.175**	**5.69**	**1.667**	**0.46**	**36.605**

a) The unbound fractions were concentrated to a volume of 1 ml, at which the UV absorbance was measured at 488 nm.

b) EGFP concentration was calculated using the standard curve, as shown in Figure 3H.

c) EGFP recovery (%) = EGFP concentration/0.3 × 100.

d) The bound fractions were concentrated to a volume of 1 ml, at which the UV absorbance was measured at 488 nm.

EGFP only (300 μg of EGFP and 100 μl of solution A) was depleted every 20th run from runs 21 to 201. Ten chromatograms were analyzed to calculate retention time and peak area. All values were determined as in Table 1. The C.V. (%) of the peak area in bound fraction was high (36.6%), caused by variations in the 10 runs. These variations in peak area might have been due to the peaks at 36 min, which resulted from the buffer components.

### Recovery of depletion using EGFP as a flow-through protein indicator

We examined the recovery of EGFP after the depletion runs. The amount of EGFP that was spiked into the plasma sample was adjusted to 300 μg. The volumes of the EGFP-containing unbound fraction and bound fraction were 2.5 ml (500 μl per vial, total 5 vials) and 5.0 ml (total 10 vials) after depletion, respectively. The volumes of the 2 fractions were reduced to 1 ml by Centricon, and the UV absorbance at 488 nm was measured using 200 μl of each concentrated sample to estimate the concentration of EGFP, based on the standard curve (Figure 3). From the concentrations of both fractions, the amounts of recovered EGFP in the unbound and bound fractions were calculated. The resulting EGFP quantities were compared with 300 μg of input EGFP, wherein the recovery of EGFP can be estimated in the unbound fraction after depletion.

The depletion recoveries using EGFP-spiked plasma and EGFP-only were summarized every 20th run in Tables 1 and 2. The average recovery of EGFP was 94.2% (1.7% of C.V.) for EGFP-spiked plasma (Table 1) and 93.8% (2.6% of C.V.) for EGFP-only (Table 2). Further, EGFP was detected only in the unbound fraction; the bound fraction contained no traces of EGFP, confirmed by SDE-SDS PAGE (Figure 5D). The loss in EGFP for EGFP-spiked plasma (5.8%, 17.4 μg) and EGFP-only (6.2%, 18.6 μg) might be attributed to accumulated errors that were caused by several tube transfers and volume reduction steps by Centricon. For these losses, EGFP did not appear to remain in the MARS column after depletion, because the recovery rate of EGFP was reproducible for the 6 consecutive plasma depletion runs in the previous section (Additional file 1: Supplementary Table S1).

### Efficiency of depletion using FITC-HSA as an indicator of high-abundance proteins

**Figure 6:**
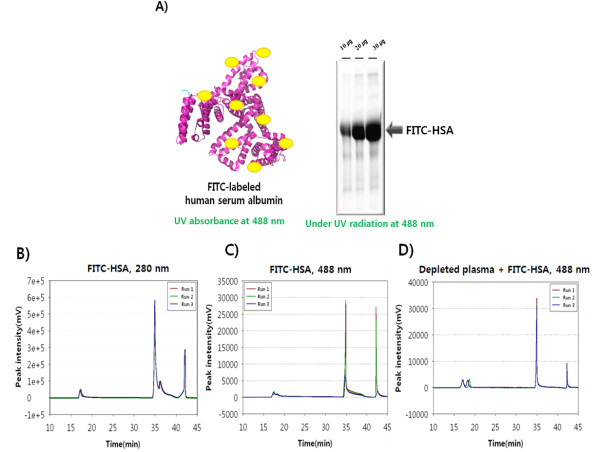
**Depletion efficiency using FITC-labeled albumin as a high-abundance protein indicator**. (A) Structural model of FITC-HSA, drawn using Pymol software [24]. Using a protein databank file, HSA (PDB ID: 1E7I) was processed to be single molecule, and the backbone of HSA was drawn in cartoon mode in magenta. Yellow circles indicate FITC on lysine residues. FITC-HSA (10, 20, and 30 μg) was subjected to SDS-PAGE (12%, 7 cm) and visualized under UV_488 nm _illumination without any staining. There were non-HSA protein bands in the lane, which were considered to be HSA-binding proteins. (B) Three chromatograms at UV_280 nm _were overlain in the three repeated depletion runs using FITC-HSA as the high-abundance protein indicator. (C) Three chromatograms at UV_488 nm _were overlain in the three repeated depletion runs using FITC-HSA as the high-abundance protein indicator. (D) Three chromatograms at UV_488 nm _were overlain in the three repeated depletion using FITC-HSA-spiked depleted plasma. The 6 most abundant proteins (HSA, immunoglobulin G, alpha-1-antitrypsin, immunoglobulin A, transferrin, and haptoglobin) were depleted in prior and 600 μg of FITC-HSA was used to spike depleted plasma, which was intended to simulate real plasma. In (B), (C), and (D), there were small unbound peaks at 17 min, which were considered to be the unbinding of FITC-HSA caused by epitopes blocking.

To examine the ability of the MARS column to capture high-abundance proteins, FITC-HSA was used as an indicator of high-abundance proteins. FITC is detected optimally at UV_488 nm _and is used as an excellent protein-labeling reagent. After purified HSA was labeled with FITC, unlabeled, excess FITC molecules were removed by buffer exchange and size-exclusion chromatography. After SDS-PAGE using 10, 20, and 30 μg of purified FITC-HSA, the gel was exposed on a UV illuminator to assess the purity and mass of FITC-HSA, based on fluorescence intensities (Figure 6A).

Although we purified FITC-HSA by gel filtration chromatography, several minor protein bands were observed in the SDS-PAGE gel, considered to be accompanying proteins that were carried by HSA. The fluorescence intensities of 3 FITC-HSA bands showed good linearity with 3 amounts of FITC-HSA (*R*^2 ^= 0.9908); the C.V. of the fluorescence intensities for the 3 FITC-HSA bands was 5.2% (Additional file 1: Supplementary Figure 2), indicating that the quantitative linearity of fluorescence intensities was excellent, based on FITC-HSA mass.

Next, 600 μg FITC-HSA was applied onto the MARS column, and UV monitoring was performed at 280 nm and 488 nm. Three repeat depletion experiments were performed using FITC-HSA, which yielded nearly identical UV absorption spectra at 280 nm and 488 nm (Figures 6B &6C). FITC-HSA, bound to the MARS column, was eluted at a retention time of 35 min. There was a small peak at 17 min, which we considered to be unbound FITC-HSA (Figures 6B &6C), which might be attributed to the unbound FITC-HSA that is caused by epitopes blocking, which may be further described (Additional file 1: Supplementary Figure 3). Two peaks at 36 min and 42 min did not contain proteins, because they were also detected in the blank run, as previously discussed.

To determine whether FITC-HSA is useful as a high-abundance protein indicator in plasma, 6 high-abundance proteins, including HSA, were depleted from plasma using the MARS column, and the depleted plasma was mixed with 600 μg FITC-HSA--an amount that presumably equals the HSA content in 16 μl plasma, an identical volume in this study of which the concentration was assumed to be 70 μg and the percentage of HSA to be 50%. HSA was chosen as a representative of 6 high-abundance proteins, because HSA is the most abundant protein; thus, it can be inferred that HSA capture affects depletion quality preferentially.

The depleted plasma, containing FITC-HSA, was applied onto the MARS column, and UV monitoring at 488 nm was performed 3 times (Figure 6D). The elution patterns of 3 repeat runs were similar overall, whereas the small peak between 18 min and 19 min was delayed slightly in the second run compared with the first and third (Figure 6D); it was ultimately considered to be a minor variation. The peak at the retention time of 17 min in both experiments (FITC-HSA in Figures 6B &6C and depleted plasma containing FITC-HSA, Figure 6D) reflected small amounts of unbound FITC-HSA. Because there were no peaks in the unbound fraction in the blank run (data not shown), we speculated that this phenomenon was caused by epitopes blocking in HSA by FITC labeling, because MARS column function through antibody affinity.

When FITC is conjugated to HSA, the ability of the MARS column resin to capture might decrease. Tagging HSA with UV-detectable fluorescent proteins, such as RFP (red) and CFP (blue), instead of FITC, might increase the efficiency of monitoring the depletion of high-abundance proteins.

### Use of EGFP and FITC-HSA saves time and costs

EGFP can be cloned, expressed in an *E. coli *expression system, and purified by NI-NTA affinity easily. A 1-L culture of *E. coli *can provide 50 mg of purified EGFP (data was not shown), which can be used at least 150 times (300 μg per reaction); these steps cost under 300 USD (for PCR-related products, LB media for *E. coli *culture, NI-NTA resin, and imidazol for purification), and EGFP preparation takes a week at most. If EGFP can be supplied as a purified solution form or powder, more time can be saved. The price of FITC powder (1 g) is approximately 200 USD, which can label 172 g HSA to be used at least 280,000 times (600 μg per reaction). HSA powder can be purchased readily and costs approximately 280 USD.

FITC-HSA takes most 4 days to prepare, and using EGFP and FITC-HSA renders the monitoring of depletion processes in real time possible. In contrast, if an ELISA kit or Western blot assay that has high sensitivity but is not online or in real time is used to monitor depletion, the initial costs (ELISA reader, etc.) might be higher, and more time will be spent for post-monitoring of depletion process. With regard to convenience and cost, EGFP and FITC have an immeasurable advantage.

The percentage of EGFP that was spiked into plasma is 11.4% (300 μg EGFP in 2.62 mg of the plasma and EGFP mixture), the level of middle abundance proteins in plasma. Since it does not require a mass spectrometer, EGFP-spiked plasma can be used periodically for quality monitoring. In addition, this quantity of EGFP will not interfere significantly with the MS analysis of low-abundance plasma proteins. However, it will be recommended that EGFP would be used to monitor flow-through proteins during depletion rather than low-abundance proteins.

Based on this study, we recommend monitoring the efficiency of depletion during the warranted use by the column supplier, using EGFP and FITC-HSA to indicate flow-through proteins and high-abundance proteins, respectively. These indicators can also be used for online monitoring that differentiates normal and abnormal depletions to allow one to remove poor batches or replace the MARS column, as described in Additional file 1: Supplementary Figure 4. FITC-HSA was used as a representative of high-abundant blood proteins that bind to the MARS column, but one abundant protein might not be enough to adequately evaluate the performance of the column. Therefore, we will develop our approach using additional abundant proteins such as alpha-1 antitrypsin or immunoglobulins in the MARS column.

## Conclusions

We adopted EGFP as a flow-through protein indicator and FITC-HSA as a high-abundance protein indicator to monitor the recovery and efficiency during depletion of high-abundance proteins from plasma or serum. This system can also be applied to other high-abundance proteins depletion systems. EGFP can be generated easily from cloned EGFP, and FITC-HSA is also readily obtainable. HPLC systems are normally equipped with a UV detector that can monitor UV absorbance of EGFP and FITC-HSA, obviating the need to determine the recovery and efficiency postexperimentally using expensive antibody-based tools, such as ELISA and Western blot. One can determine the efficiency of recovery and reproducibility online during depletion conveniently in this system.

Trivial amounts of FITC-HSA were not captured, possibly due to the blocking of antibody epitopes by the FITC-labeled moiety, but these constant unbinding of FITC-HSA were small and taking into these fact into consideration would make analysis more accountable. To overcome this phenomenon, we are attempting to fuse fluorescent proteins with serum albumin, transferrin, and antitrypsin as additional indicators of high-abundance proteins. Although we have used only the MARS column as a model system, our results suggest that the 2 indicator proteins EGFP and FITC-labeled albumin can be used conveniently to monitor the recovery and efficiency of depletion from plasma or serum using other types of immunoaffinity columns. In biomarker discovery, this process maintains the quality in hundreds of plasma depletion runs.

## Competing interests

The authors declare that they have no competing interests.

## Authors' contributions

KK and JY collected clinical plasma samples, performed the proteomics experiments, and wrote the manuscript. HK and BK performed liquid chromatography purification. HM analyzed the MS data statistics. HGY designed the clinical experiments and oversaw the IRB approval procedure. YK supervised the work, provided useful suggestions to improve performance, and revised the manuscript. All authors have read and approved the manuscript.

## Supplementary Material

Additional file 1 **Supplementary data**. Four supplementary figures and one table were included. Click here for file

## References

[B1] Pieper R Su Q Gatlin CL Huang ST Anderson NL Steiner S Multi-component immunoaffinity subtraction chromatography: an innovative step towards a comprehensive survey of the human plasma proteome Proteomics 2003 3 422 432 10.1002/pmic.200390057 12687610

[B2] Bjorhall K Miliotis T Davidsson P Comparison of different depletion strategies for improved resolution in proteomic analysis of human serum samples Proteomics 2005 5 307 317 10.1002/pmic.200400900 15619298

[B3] Echan LA Tang HY Ali-Khan N Lee K Speicher DW Depletion of multiple high-abundance proteins improves protein profiling capacities of human serum and plasma Proteomics 2005 5 3292 3303 10.1002/pmic.200401228 16052620

[B4] Zolotarjova N Martosella J Nicol G Bailey J Boyes BE Barrett WC Differences among techniques for high-abundance protein depletion Proteomics 2005 5 3304 3313 10.1002/pmic.200402021 16052628

[B5] Huang L Harvie G Feitelson JS Gramatikoff K Herold DA Allen DL Amunngama R Hagler RA Pisano MR Zhang WW Fang X Immunoaffinity separation of plasma proteins by IgY microbeads: meeting the needs of proteomic sample preparation and analysis Proteomics 2005 5 3314 3328 10.1002/pmic.200401277 16041669

[B6] Yocum AK Yu K Oe T Blair IA Effect of immunoaffinity depletion of human serum during proteomic investigations J Proteome Res 2005 4 1722 1731 10.1021/pr0501721 16212426

[B7] Brand J Haslberger T Zolg W Pestlin G Palme S Depletion efficiency and recovery of trace markers from a multiparameter immunodepletion column Proteomics 2006 6 3236 3242 10.1002/pmic.200500864 16645986

[B8] Bellei E Bergamini S Monari E Fantoni LI Cuoghi A Ozben T Tomasi A High-abundance proteins depletion for serum proteomic analysis: concomitant removal of non-targeted proteins Amino Acids 20495836 10.1007/s00726-010-0628-x

[B9] Tu C Rudnick PA Martinez MY Cheek KL Stein SE Slebos RJ Liebler DC Depletion of Abundance Plasma Proteins and Limitations of Plasma Proteomics J Proteome Res 2010 9 4982 4991 10.1021/pr100646w 20677825 PMC2948641

[B10] Polaskova V Kapur A Khan A Molloy MP Baker MS High-abundance protein depletion: comparison of methods for human plasma biomarker discovery Electrophoresis 31 471 482 10.1002/elps.200900286 20119956

[B11] Chard MD Calvin J Price CP Cawston TE Hazleman BL Serum alpha 1 antichymotrypsin concentration as a marker of disease activity in rheumatoid arthritis Ann Rheum Dis 1988 47 665 671 10.1136/ard.47.8.665 3261967 PMC1006719

[B12] Sitnikov D Chan D Thibaudeau E Pinard M Hunter JM Protein depletion from blood plasma using a volatile buffer J Chromatogr B Analyt Technol Biomed Life Sci 2006 832 41 46 10.1016/j.jchromb.2005.12.013 16414315

[B13] Zhang G Gurtu V Kain SR An enhanced green fluorescent protein allows sensitive detection of gene transfer in mammalian cells Biochem Biophys Res Commun 1996 227 707 711 10.1006/bbrc.1996.1573 8885998

[B14] Yang TT Cheng L Kain SR Optimized codon usage and chromophore mutations provide enhanced sensitivity with the green fluorescent protein Nucleic Acids Res 1996 24 4592 4593 10.1093/nar/24.22.4592 8948654 PMC146266

[B15] Cormack BP Valdivia RH Falkow S FACS-optimized mutants of the green fluorescent protein (GFP) Gene 1996 173 33 38 10.1016/0378-1119(95)00685-0 8707053

[B16] Wang Y Cheung YH Yang Z Chiu JF Che CM He QY Proteomic approach to study the cytotoxicity of dioscin (saponin) Proteomics 2006 6 2422 2432 10.1002/pmic.200500595 16548062

[B17] Lelong C Chevallet M Luche S Rabilloud T Silver staining of proteins in 2DE gels Methods Mol Biol 2009 519 339 350 full_text 19381593 10.1007/978-1-59745-281-6_21

[B18] Park J Kwon H Kang Y Kim Y Proteomic analysis of O-GlcNAc modifications derived from streptozotocin and glucosamine induced beta-cell apoptosis J Biochem Mol Biol 2007 40 1058 1068 18047804 10.5483/bmbrep.2007.40.6.1058

[B19] Jin M Protocol for FITC labeling of proteins 2007 http://jin-laborg/wiki/protocols/fitc_labeling_of_proteins

[B20] Lei T He QY Wang YL Si LS Chiu JF Heparin chromatography to deplete high-abundance proteins for serum proteomics Clinica Chimica Acta 2008 388 173 178 10.1016/j.cca.2007.10.034 18036563

[B21] Freeman WM Lull ME Guilford MT Vrana KE Depletion of abundance proteins from non-human primate serum for biomarker studies Proteomics 2006 6 3109 3113 10.1002/pmic.200500717 16619306

[B22] Petricoin EF Liotta LA SELDI-TOF-based serum proteomic pattern diagnostics for early detection of cancer Curr Opin Biotechnol 2004 15 24 30 10.1016/j.copbio.2004.01.005 15102462

[B23] Zhou M Lucas DA Chan KC Issaq HJ Petricoin EF Liotta LA Veenstra TD Conrads TP An investigation into the human serum "interactome" Electrophoresis 2004 25 1289 1298 10.1002/elps.200405866 15174051

[B24] Ordog R PyDeT, a PyMOL plug-in for visualizing geometric concepts around proteins Bioinformation 2008 2 346 347 18685723 10.6026/97320630002346 PMC2478735

