# Supplementary material for: Online monitoring of immunoaffinity-based depletion of high-abundance blood proteins by UV spectrophotometry using enhanced green fluorescence protein and FITC-labeled human serum albumin
Source: Proteome Sci. 2010 Dec 1;8:62. doi: 10.1186/1477-5956-8-62 (PMC3012035; doi:10.1186/1477-5956-8-62)
Supplement: Additional file 1 — Supplementary data. Four supplementary figures and one table were included. [file 1477-5956-8-62-S1.DOC]

**Supplementary Figure 1. Determination of labeling efficiency for FITC-HSA**


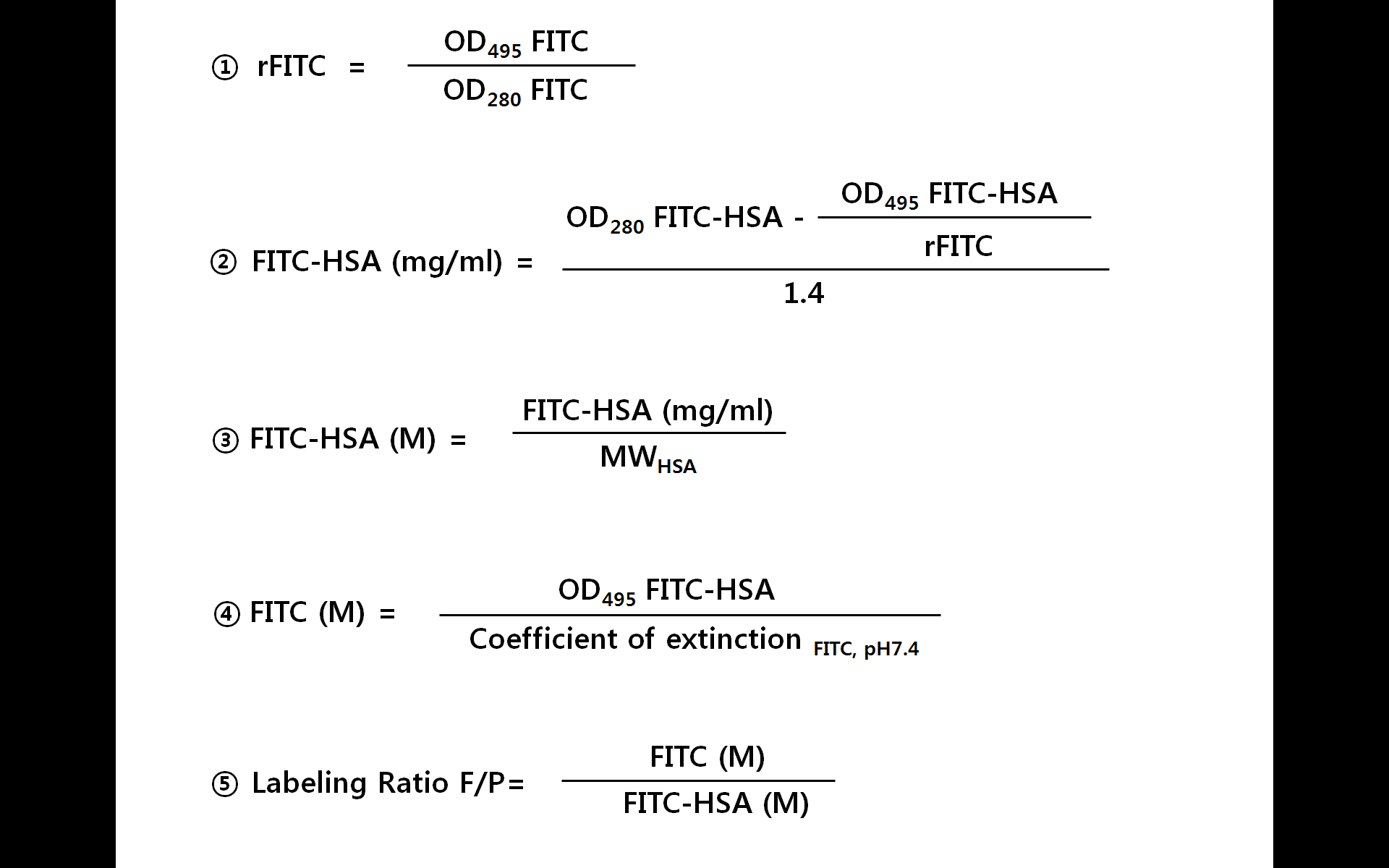


FITC is an excellent protein labeling reagent. In basic buffer, the isothiocyanate reactive group (-N=C=S) of FITC can form bonds with amine and sulfhydryl groups on proteins. In this study, the ratio of FITC to HSA was intended to be 2.56:1, as suggested by the Jin Lab (http://jin-lab.org/wiki/protocols/fitc_labeling_of_proteins). The labeling procedure is described in the Methods section, and the labeling ratio of FITC to HSA was calculated according to the 5 sequential equations above.

The UV absorbance of FITC and FITC-HSA at 495 nm and 280 nm was measured 5 times on a NanoDrop spectrophotometer (model number: ND-1000, NanoDrop Technologies, Inc., USA). All measured values were averaged, generating OD_495_ (UV absorbance at 495 nm) and OD_280_ (UV absorbance at 280 nm) values for FITC of 0.6443 and 0.1949, respectively. The rFITC was 3.3. The OD_495_ and OD_280_ values of FITC-HSA were 0.10034 and 0.8705, respectively. The molecular weight of mature HSA (MW_HSA_) is 66472.2 Da, and the extinction coefficient of FITC at pH 7.4 is 63,000. Therefore, the labeling ratio of FITC to HSA (Labeling Ratio F/P) was calculated to be 2.61, which is close to the input ratio of 2.56, indicating that input FITC was labeled nearly 100% of the HSA, leaving little unlabeled HSA.

**Supplementary Figure 2. Amounts of FITC-HSA correlate with fluorescence intensities of FITC**


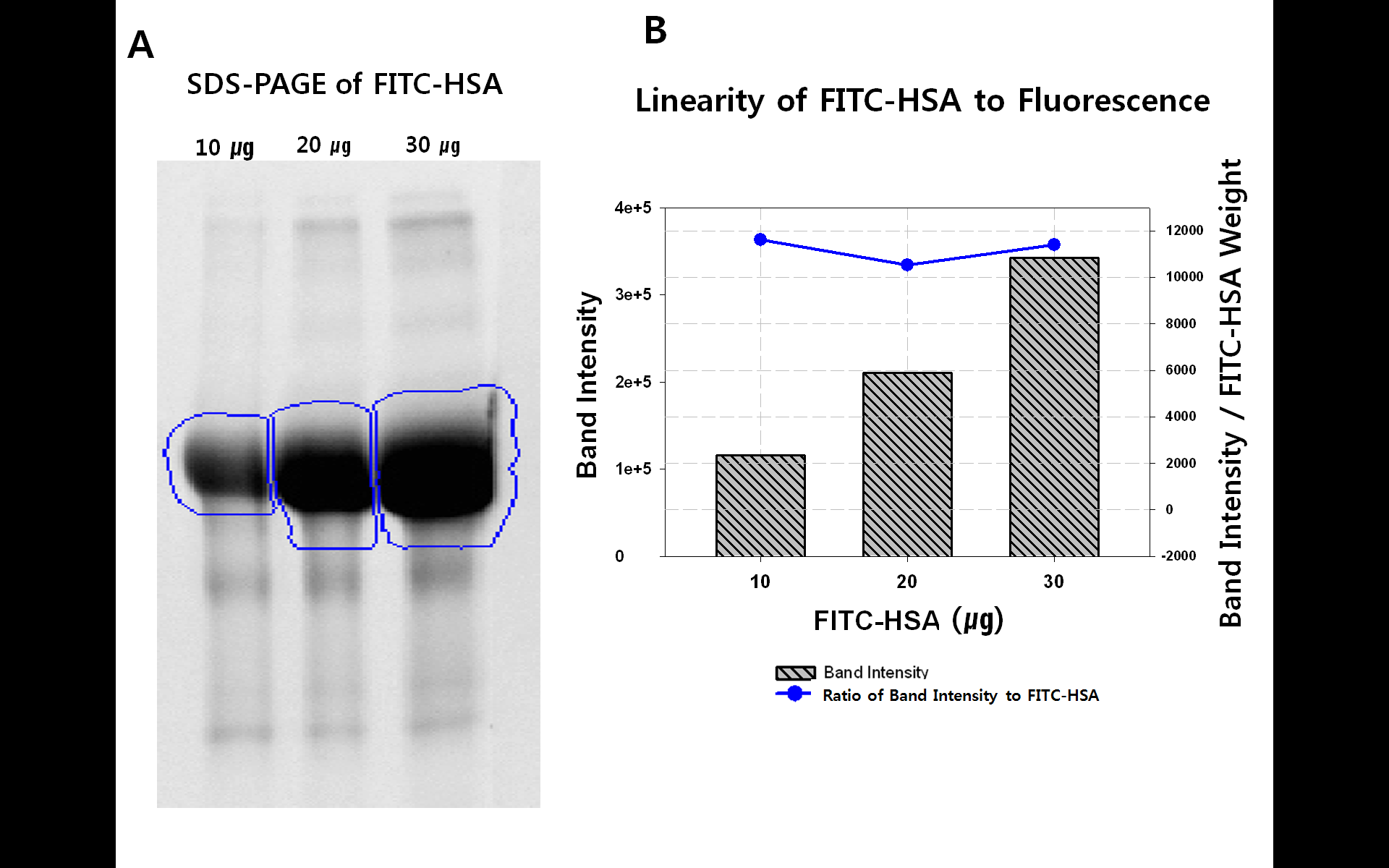


Ten, twenty, and thirty micrograms of FITC-HSA, purified using gel filtration chromatography, was subjected to SDS-PAGE. The gel was exposed to a UV illuminator (Supplementary Figure 2A) without staining. In the UV-exposed gel, FITC-labeled proteins luminesced, and the FITC-HSA band was largest band; there were several minor bands that were considered to be HSA-binding proteins.

HSA-binding proteins can interfere with the fluorescence linearity from FITC-HSA. To assess the fluorescence linearity by input FITC-HSA, the band intensities of FITC-HSA were calculated using densitometry software (Phoretix 2D Expression program, Nonlinear Dynamics, Durham, NC). The background was subtracted using the “mode of nonspot” tool, and its margin value was set to 45. The spot margin around the bands is shown as a blue line in Supplement Figure 2A. The band intensities of 10, 20, and 30 μg FITC-HSA were 116147.6, 210323.4, and 342160.9, respectively. The correlation between FITC-HSA weight and band intensity was *R^2^* =0.9908. The band intensities per 1 μg FITC-HSA in three lanes were 11614.8, 10516.2, and 11405.4, respectively, showing 5.2% of the C.V. In Supplementary Figure 2B, the bar-shaped graph represents the intensities for 3 amounts of FITC-HSA, and the blue line indicates the band intensity-versus-FITC-HSA weight ratio, demonstrating that FITC-HSA fluorescence shows good linearity against FITC-HSA amounts.

**Supplementary Figure 3. Minute amounts of FITC-HSA do not bind to the MARS column**
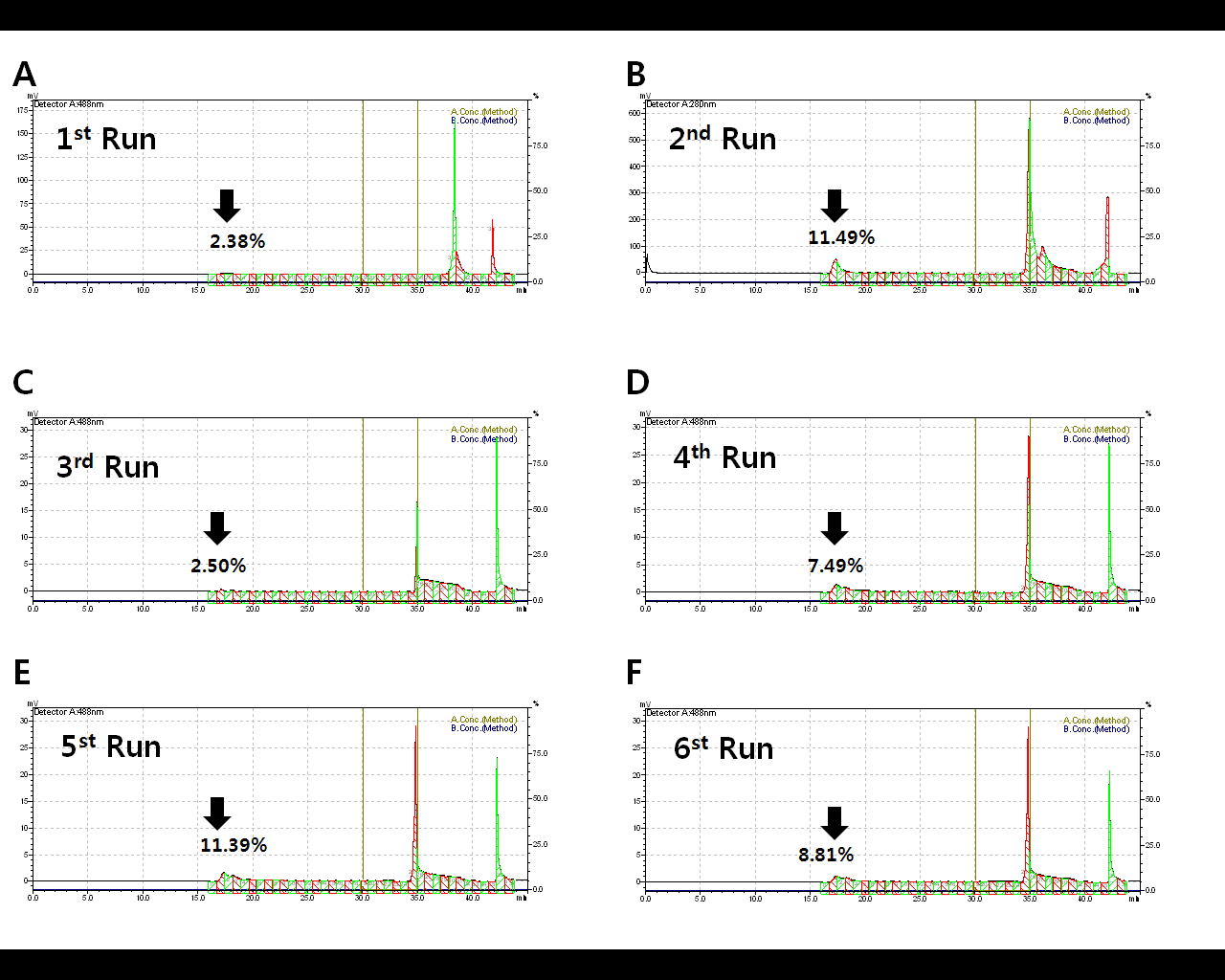


Six repeat depletions of FITC-HSA only (600 μg) were performed using the MARS column, with UV absorbance monitoring at 488 nm (A - F). The major peaks at 35 min in all 6 chromatograms represent FITC-HSA, which was captured on the MARS column and eluted by urea-based solution B. The peaks at 43 min were from buffer components, which also appeared in the blank run. The minor peaks at 17 min, indicated by black arrows, were considered to be uncaptured FITC-HSA. The number below the arrows is the percentage of peak area. The minor peak at 17 min appeared consistently in the 6 runs, and the variation in peak area ranged from 2.38% to 11.39%.

The MARS column is based on antibody-affinity capture of target proteins. Although FITC versus HSA was intended to be at molar ratio of 2.5:1, the FITC labeling of amino acids on the protein's surface, particularly the eta-amine of lysine and alpha-amine of N-terminal amino acids, is random and uncontrollable. Conceivably, antibody epitopes of HSA might also be labeled with FITC, which might prevent FITC-HSA from binding to the antibodies on the MARS column resin. Regardless, these unbound FITC-HSA peaks were relatively small compared with the bound FITC-HSA; thus, they do not impair the monitoring of plasma depletion.

**Supplementary Figure 4. FITC-HSA as an indicator of high-abundance proteins during depletion**


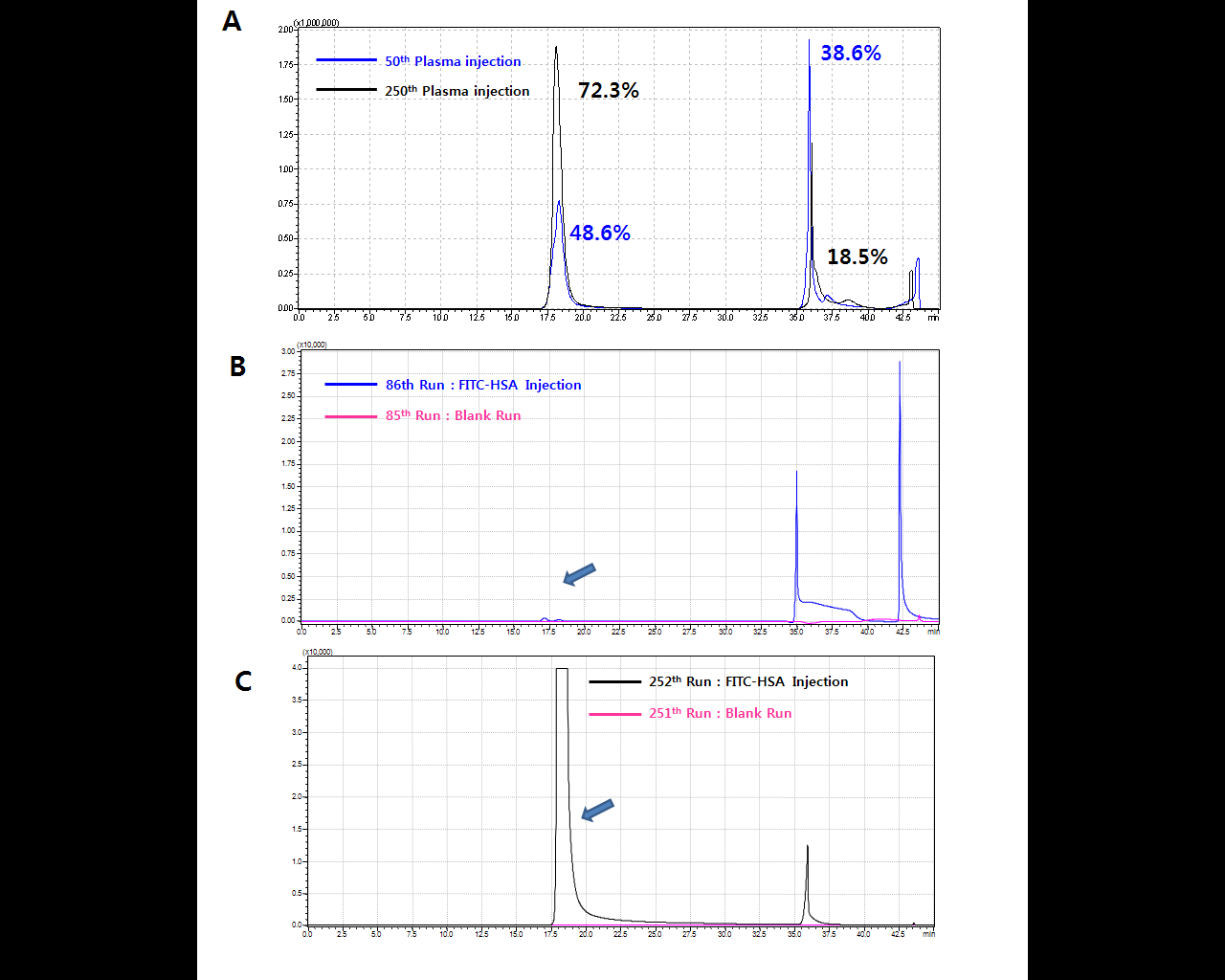


In Supplementary Figure 4A, 2 UV absorbance chromatograms at 280 nm were overlain. The blue line was obtained from the 50th depletion, and the black line was the 250th depletion using 100 μl of 5-fold-diluted control plasma (approximately 1.6 mg). Numbers represent percentage of peak area compared with the total peak area.

During repetitive depletion runs, antibodies on the MARS column can deteriorate, decreasing the capture capacity. As the number of depletion experiments increases, the peak area of the unbound fraction increases, while that of the bound fraction from high-abundance proteins decreases, as shown in Supplementary Figure 4A. In fact, the difference between the 2 runs (50th and 250th runs) was detected easily, because we used the same control samples subsequently, however, the difference that was caused by deterioration of the MARS column or abnormal operation might not be recognized easily by visual inspection without control experiments.

In Supplementary Figure 4B, the chromatograms for 2 depletion experiments using FITC-HSA only (blue) and blank (pink) are overlain (86th and 85th run, respectively) by UV monitoring at 488 nm. FITC-HSA was bound to the MARS column and eluted at 35 min, and FITC-HSA was the major peak, while a small amount of unbound FITC-HSA constituted the peak at 17 min (blue arrow).

In contrast, in the chromatogram of the 252th experiment that was performed under identical conditions as in the 86th run (Supplementary Figure 4C), the unbound FITC-HSA formed the major peak at 17.5 min, and the peak at 35 min decreased compared with the pattern of the 86^th^ run. The 252th run with FITC-HSA as the high-abundance protein indicator will provide more dramatic signal with regard to monitoring compared with UV monitoring at 280 nm as in the 250^th^ run, because at 280 nm, the total amount of eluted proteins is monitored, while at 488 nm, only high-abundance proteins, including FITC-HSA, are monitored.

**Table S1. Recovery using EGFP-spiked plasma in 6 consecutive depletion runs**

| **Run** | **O.D_488nm_^a)^** | **EGFP conc.** | **Recovery** | **Retention Time** | **Peak Area** |
| --- | --- | --- | --- | --- | --- |
|  |  | **(mg/ml)^b)^** | **(%)^c)^** | **(minute)** |  |
| **1** | 0.323 | 0.294 | 97.869 | 17.153 | 42853087 |
| **2** | 0.313 | 0.283 | 94.348 | 17.135 | 42241736 |
| **3** | 0.323 | 0.294 | 97.869 | 17.133 | 42173830 |
| **4** | 0.301 | 0.27 | 90.122 | 17.129 | 41879936 |
| **5** | 0.319 | 0.289 | 96.461 | 17.135 | 41974861 |
| **6** | 0.316 | 0.286 | 95.404 | 17.168 | 38653456 |
| **Average** | **0.316** | **0.286** | **95.345** | **17.142** | **41629484.33** |
| **S.D.** | **0.0083** | **0.0087** | **2.9077** | **0.015** | **1497138.839** |
| **C.V. (%)** | **2.627** | **3.042** | **3.05** | **0.088** | **3.596** |

a) The unbound fractions were concentrated to 1 ml, and UV absorbance was measured at 488 nm.

b) EGFP concentration was calculated using linear regression, shown in Figure 3.

c) EGFP recovery (%) = EGFP concentration/0.3 ⅹ 100

EGFP-spiked plasma (300 μg of EGFP and 2.62 mg of plasma in 100 μl) was depleted in 6 repetitive runs. Six chromatograms were analyzed to calculate retention time and peak area. In addition, the unbound fraction containing EGFP as the flow-through protein indicator was concentrated to 1.0 ml from 6 consequent depletions, in which OD_488 nm_ was measured to estimate recovery. Concentrations of EGFP in the unbound fraction were calculated, based on the standard curve in Figure 3. The C.V. (%) value was calculated such that the standard deviation was divided by the average and multiplied by 100.
